# High yield 1,3-propanediol production by rational engineering of the 3-hydroxypropionaldehyde bottleneck in *Citrobacter werkmanii*

**DOI:** 10.1186/s12934-016-0421-y

**Published:** 2016-01-28

**Authors:** Veerle E. T. Maervoet, Sofie L. De Maeseneire, Fatma G. Avci, Joeri Beauprez, Wim K. Soetaert, Marjan De Mey

**Affiliations:** Department of Biochemical and Microbial Technology, Centre of Expertise-Industrial Biotechnology and Biocatalysis, Ghent University, Coupure links 653, 9000 Ghent, Belgium; Department of Applied Bioscience Engineering, Laboratory of Applied Biotechnology, Ghent University, Valentin Vaerwyckweg 1, 9000 Ghent, Belgium; Bioengineering Department, Faculty of Engineering, Ege University, 35100 Bornova-Izmir, Turkey

**Keywords:** 1,3-propanediol, Glycerol, *Citrobacter werkmanii* DSM17579, NADH, Lactate dehydrogenase, Ethanol dehydrogenase, Multiple knock-out mutant, Rational engineering

## Abstract

**Background:**

Imbalance in cofactors causing the accumulation of intermediates in biosynthesis pathways is a frequently occurring problem in metabolic engineering when optimizing a production pathway in a microorganism. In our previous study, a single knock-out *Citrobacter werkmanii* ∆*dhaD* was constructed for improved 1,3-propanediol (PDO) production. Instead of an enhanced PDO concentration on this strain, the gene knock-out led to the accumulation of the toxic intermediate 3-hydroxypropionaldehyde (3-HPA). The hypothesis was emerged that the accumulation of this toxic intermediate, 3-HPA, is due to a cofactor imbalance, i.e. to the limited supply of reducing equivalents (NADH). Here, this bottleneck is alleviated by rationally engineering cell metabolism to balance the cofactor supply.

**Results:**

By eliminating non-essential NADH consuming enzymes (such as lactate dehydrogenase coded by *ldhA*, and ethanol dehydrogenase coded by *adhE*) or by increasing NADH producing enzymes, the accumulation of 3-HPA is minimized. Combining the above modifications in *C. werkmanii* ∆*dhaD* resulted in the strain *C. werkmanii* ∆*dhaD*∆*ldhA*∆*adhE*::ChlFRT which provided the maximum theoretical yield of 1.00 ± 0.03 mol PDO/mol glycerol when grown on glucose/glycerol (0.33 molar ratio) on flask scale under anaerobic conditions. On bioreactor scale, the yield decreased to 0.73 ± 0.01 mol PDO/mol glycerol although no 3-HPA could be measured, which indicates the existence of a sink of glycerol by a putative glycerol dehydrogenase, channeling glycerol to the central metabolism.

**Conclusions:**

In this study, a multiple knock-out was created in *Citrobacter* species for the first time. As a result, the concentration of the toxic intermediate 3-HPA was reduced to below the detection limit and the maximal theoretical PDO yield on glycerol was reached.

**Electronic supplementary material:**

The online version of this article (doi:10.1186/s12934-016-0421-y) contains supplementary material, which is available to authorized users.

## Background

Nicotinamide adenine dinucleotide (NAD^+^) plays a central role in the cellular metabolism of microorganisms by functioning as a cofactor in over 300 oxidation-reduction reactions [1]. Moreover, the NADH/NAD^+^ ratio has a regulatory effect on the expression of some genes and the activity of certain enzymes. For example, a high NADH/NAD^+^ ratio results in an enhanced expression of the ethanol dehydrogenase AdhE [2], which catalyzes the formation of ethanol during fermentation, and increases the inhibition of the pyruvate dehydrogenase complex [3, 4]. Thus, reaching cofactor balance is important for a high titer, yield and rate in the biosynthesis of interesting metabolites.

During the production of 1,3-propanediol (PDO) from glycerol as sole carbon source in natural PDO producing strains such as the opportunistic pathogen *Citrobacter werkmanii*, the cofactors are balanced via the two glycerol dissimilation pathways (Additional file 1: Figure S1). Via the oxidative branch, glycerol dehydrogenase (GDH, E.C. 1.1.1.6), encoded by *dhaD*, forms NADH which, in turn, is consumed by 1,3-propanediol dehydrogenase (PDODH, E.C. 1.1.1.202), coded by *dhaT* in the reductive branch [2]. Indeed, in our previous work an imbalance in the PDO production was observed in *C. werkmanii* DSM17579 due to the deletion of *dhaD* [5]. The cofactor/pathway imbalance not only resulted in the accumulation of the intermediate 3-hydroxypropionaldehyde (3-HPA) but also in growth inhibition and incomplete PDO production before substrate depletion.

Several strategies have been reported to balance the cofactors, to reduce the buildup of 3-HPA and thus to increase the yield of PDO on glycerol. Among them, the most important are cofactor regeneration, elimination of non-essential NADH-consuming enzymes, and promotion of the activity of NADH producing enzymes. Zhang et al. [6] and Luo et al. [7] have increased the yield of PDO on glycerol using an in vivo cofactor regeneration system which converts NAD^+^ into NADH, while, respectively, CO_2_ is produced from formate by formate dehydrogenase, and 3-HPA is changed into 3-hydroxypropionic acid by an aldehyde dehydrogenase AldH. For the deletion of the genes which products consume NADH, the focus was laid on the lactate dehydrogenase gene *ldhA* in *Klebsiella oxytoca* M5a1 [8] and *K. pneumoniae* HR526 [9] and on an aldehyde dehydrogenase gene *aldA* in *K. pneumoniae* YMU2 [10]. In both cases one NADH is consumed per reaction. Deletion of the genes resulted in a significantly enhanced NADH/NAD^+^ ratio, and consequently an increased yield on glycerol of PDO and improved final PDO titer in *Klebsiella* sp. [8–11].

The last method to increase the intracellular NADH concentration, enhancement of NADH producing enzymes, is attained by manipulating the citric acid cycle (TCA cycle) and its regulation. Most NADH of the TCA cycle is produced under aerobic conditions: four NADH molecules are formed per molecule glucose. However, under anaerobic conditions, which are the circumstances of PDO production, only a reductive branched TCA cycle is active, which consumes, instead of produces, NADH (Additional file 1: Figure S2) [12]. Hence, to increase the production of NADH, the regulation of the TCA cycle should be altered to an aerobic arrangement under anaerobic conditions. To accomplish this, the phosphorylation of ArcA, one of the global regulators, should be avoided through deletion of *arcA* or *arcB*. This deletion has been used by several researchers, for example, to increase the activity of NADH-consuming reactions for the production of succinate and poly[(R)-3-hydroxybutyrate] [13–15]. However, the effect of this deletion on PDO production was never investigated before.

In this study, the last two approaches are tested (Additional file 1: Figure S1): deletion of the genes *ldhA* and *adhE*, coding for lactate dehydrogenase and ethanol dehydrogenase, respectively, and enhancement of the NADH production by deletion of *arcA*. The different single and multiple knock-out mutants of *C. werkmanii* DSM17579 are evaluated on the growth, PDO yield, final PDO concentration and NAD^+^/NADH ratio. The best performer is evaluated in batch fermentation at a higher concentration of glycerol. To the best of our knowledge, this is the first time the effect of *arcA* on PDO will be studied. Moreover, most research has been performed on improving PDO production using a single knock-out mutant in the pathogenic *Klebsiella* species. The (possible synergistic) effect(s) of combining a split of the glycerol metabolizing pathway and deletion of byproduct formation has barely been investigated.

## Results and discussion

In order to test the hypothesis on cofactor improvement for minimizing the intermediate accumulation and maximizing PDO production in *C. werkmanii* ∆*dhaD* several single mutants were rationally designed and evaluated. Further, these mutants were recombined in a single strain, which significantly minimizes the 3-HPA accumulation and allows the strain to reach maximum PDO production yields.

### Construction and evaluation of single knock-out mutants

Based upon previously reported strategies to improve cofactor availability in other microorganisms via metabolic engineering, three genes were selected to knock-out and to evaluate towards their effect on viability and production of 1,3-propanediol in *C. werkmanii* DSM17579. Two non-essential enzymes consuming NADH (LdhA, AdhE), and one global regulator, ArcA, which represses reactions producing NADH were eliminated, respectively resulting in the single gene knock-outs *C. werkmanii ∆ldhA, C. werkmanii ∆adhE::ChlFRT, and C. werkmanii ∆arcA.* After elucidation of the gene sequences, the mutants were constructed using an in-house gene deletion technique developed for *C. werkmanii* [5]. The sequence results of the different knock-outs are presented in Additional file 2.

To investigate the effect of the single knock-outs on the growth and metabolic profile of *C. werkmanii* DSM17579, the different mutants were grown in shake flasks with 163 mM glycerol as sole carbon source under anaerobic conditions. As depicted in Table 1, the growth rate decreases for all mutants compared to the wild-type. For *C. werkmanii* ∆*arcA* the growth rate is halved. The decline is consistent with the findings of Zhang et al. [10] and Nizam and Shimizu [16] for the single knock-out of, respectively, *aldH* in *K. pneumoniae* YMU2 and *arcB* in *Escherichia coli* BW25113. They suggest that the accumulation of pyruvate around the pyruvate node, caused by these mutations, may prompt dumping of the glycolysis flux, resulting in a reduced growth rate [16].

**Table 1:** Growth rate, PDO yield and metabolite profile of the wild-type (WT) and single knock-outs

Strains	Growth rate (h^−1^)	PDO Yield (mol/mol)	Metabolite concentration (mM)			
			Acetate	Ethanol	Lactate	Succinate
WT	0.33 ± 0.02	0.63 ± 0.01	38.20 ± 0.83	7.08 ± 0.37	1.14 ± 0.27	4.69 ± 0.14
∆*ldhA*	0.28 ± 0.02	0.59 ± 0.02	18.30 ± 1.07	0.21 ± 0.14	BDL	4.72 ± 0.08
∆*adhE*::ChlFRT	0.26 ± 0.01	0.70 ± 0.01	44.30 ± 2.19	BDL	4.67 ± 0.31	5.93 ± 0.09
∆*arcA*::ChlFRT	0.13 ± 0.00	0.65 ± 0.01	58.00 ± 1.30	BDL	5.27 ± 0.84	7.09 ± 0.32

*C. werkmanii* DSM17579 (WT), *C. werkmanii* ∆*ldhA*, *C. werkmanii* ∆*adhE*::ChlFRT, and *C. werkmanii* ∆*arcA*::ChlFRT were cultivated under anaerobic conditions in shake flasks. The values are the average of two experiments with their corresponding standard deviations

*BDL* below the detection limit

Only for *C. werkmanii* ∆*adhE* a higher PDO yield is obtained. The 11.00 ± 2.25 % increase in PDO yield on glycerol is probably achieved because four additional NADH molecules become available per glucose molecule after deleting the *adhE* gene. These NADH molecules can be used by PDODH to convert 3-HPA into PDO. The abolished ethanol production in *C. werkmanii* ∆*adhE* indicates that the knock-out is successful. Thereby, more acetyl-CoA is available for acetate formation, resulting in an increased acetate production by this knock-out. The final succinate and especially lactate titer are enhanced as well in *C. werkmanii* ∆*adhE*. Similar results were obtained by Zhang et al. [10] who deleted an aldehyde dehydrogenase gene in *K. pneumoniae* YMU2: the acetate, lactate, 2,3-butanediol, and PDO production increased, while the succinate production decreased.

An analogous redistribution of the metabolic fluxes is observed when growing *C. werkmanii* ∆*arcA*. An increase in all measured metabolites is observed, except for ethanol, which is reduced below the detection limit. Previous studies have shown that deletion of *arcA* results in an elevated expression of the TCA cycle genes, an improved NADH/NAD^+^ ratio, and an elevated final concentration of NADH consuming metabolites, such as lactate and succinate [17]. Nizam and Shimizu [16] describe the inactivation of ArcB, the sensor of the Arc system, in *Escherichia coli* BW25113. Deletion of this gene results in unphosphorylated ArcA under anaerobic conditions, which causes increased D-lactate concentration, and a decreased acetate, ethanol and formate concentration. They correlate these findings to a reduced flux through pyruvate-formate lyase (E.C. 2.3.1.54). In our experiments, the ethanol concentration is also reduced, whereas the acetate concentration is enhanced. Therefore, we ascribe the metabolic redistribution rather to the altered NADH/NAD^+^ ratio than to the decreased flux through pyruvate-formate lyase. However, enzyme assays or metabolic flux analysis should be performed to confirm this.

### Construction of double and triple mutants and evaluation of their synergetic effects

The single knock-out study revealed that only the *adhE* single knock-out has a positive effect on the yield of PDO on glycerol and that the titers of the fermentation products consuming NADH increase, especially the lactate titer in *C. werkmanii* ∆*adhE*. Therefore, combinations of the *adhE* and *ldhA* knock-outs with the *dhaD* knock-out were tested; the latter was proven beneficial in previous research [5]. Specifically, two double (*C. werkmanii* ∆*dhaD*∆*ldhA* and *C. werkmanii* ∆*dhaD*∆*adhE*) and one triple (*C. werkmanii* ∆*dhaD*∆*ldhA*∆*adhE*) mutants were constructed to investigate synergistic effects. The mutant strains were grown anaerobically in shake flasks with 40 mM glucose and 120 mM glycerol, yielding a molar ratio of 0.33 glucose/glycerol. No double mutants containing the *arcA* knock-out were constructed as *C. werkmanii* Δ*dhaD*Δ*ldhA*Δ*arcA* did not produce any 1,3-propanediol (data not shown).

Compared to the single knock-out mutant, *C. werkmanii* ∆*dhaD*, the newly constructed double mutant strains have a slightly improved growth rate and yield a significantly higher final PDO concentration and PDO yield on glycerol due to a vastly reduced NAD^+^/NADH ratio (Tables 2, 3).

**Table 2:** Growth rate, PDO yield, NAD^+^/NADH-ratio and pH of *C. werkmanii* knock-outs

Strains	Growth rate (h^−1^)	PDO Yield (mol/mol)	NAD^+^/NADH ratio	Final pH
∆*dhaD*	0.25 ± 0.01	0.68 ± 0.05	4.74 ± 0.14	6.43 ± 0.01
∆*dhaD*∆*ldhA*	0.31 ± 0.03	0.84 ± 0.01	1.66 ± 0.03	6.35 ± 0.02
∆*dhaD*∆*adhE*::ChlFRT	0.34 ± 0.01	0.96 ± 0.01	2.20 ± 0.07	6.33 ± 0.03
∆*dhaD*∆*ldhA*∆*adhE*::ChlFRT	0.13 ± 0.01	1.00 ± 0.03	2.38 ± 0.15	6.80 ± 0.00

*C. werkmanii* ∆*dhaD*, *C. werkmanii* ∆*dhaD*∆*ldhA, C. werkmanii* ∆*dhaD*∆*adhE*::ChlFRT and *C. werkmanii* ∆*dhaD*∆*ldhA*∆*adhE*::ChlFRT grown anaerobically in shake flasks. The values are the average of two experiments with their corresponding standard deviation

**Table 3:** Residual substrate concentration and metabolite profile of *C. werkmanii* knock-outs

Strains	Residual concentration (mM)		Metabolite concentration (mM)					
	Glycerol	Glucose	PDO	Acetate	Ethanol	Lactate	Succinate	3-HPA
∆*dhaD*	45.52 ± 1.56	0.22 ± 0.03^a^	39.80 ± 2.67^a^	46.20 ± 0.59^a^	18.00 ± 1.22^a^	3.10 ± 0.49^a^	4.44 ± 0.30^a^	24.74 ± 2.48^a^
∆*dhaD*∆*ldhA*	41.92 ± 0.89	2.75 ± 0.20^b^	62.90 ± 1.29^a,b^	52.40 ± 0.99^a,b^	12.50 ± 0.21^a,b^	0.87 ± 0.02^a,b^	5.11 ± 0.02^b^	11.92 ± 1.35^a,b^
∆*dhaD*∆*adhE*::ChlFRT	44.43 ± 5.37	9.08 ± 2.71^a^	86.11 ± 7.19^a,b,c^	68.12 ± 1.74^a,b,c^	BDL^a,b^	5.46 ± 0.61^a,b,c^	6.36 ± 0.61^a,b^	4.83 ± 0.21^a,b,c^
∆*dhaD*∆*ldhA*∆*adhE*::ChlFRT	52.33 ± 5.63	11.05 ± 1.26^a,b^	107.20 ± 4.22^a,b,c^	75.40 ± 0.69^a,b,c^	BDL^a,b^	BDL^a,c^	6.07 ± 0.14^a^	BDL^a,b,c^

*C. werkmanii* ∆*dhaD*, *C. werkmanii* ∆*dhaD*∆*ldhA, C. werkmanii* ∆*dhaD*∆*adhE*::ChlFRT and *C. werkmanii* ∆*dhaD*∆*ldhA*∆*adhE*::ChlFRT grown anaerobically in shake flasks. The values are the average of two experiments with their corresponding standard deviation

*BDL* below the detection limit

^a, b, c^Significant values calculated by one-way ANOVA and Bonferroni post hoc test

The final PDO titer is considerably increased, from 39.80 ± 2.67 mM in the *C. werkmanii* ∆*dhaD* knock-out to 62.90 ± 1.29 mM and 86.11 ± 7.19 mM in the *C. werkmanii* ∆*dhaD*∆*ldhA* and *C. werkmanii* ∆*dhaD*∆*adhE* double knock-outs, respectively. As the residual glycerol concentration remains constant, the yield on glycerol of PDO increases to 0.84 ± 0.01 and 0.96 ± 0.01 mol PDO/mol glycerol using *C. werkmanii* ∆*dhaD*∆*ldhA* and *C. werkmanii* ∆*dhaD*∆*adhE*, respectively. Furthermore, the final 3-HPA titer is reduced significantly comparing *C. werkmanii* ∆*dhaD* (Table 3): a 50 % reduction is observed in *C. werkmanii* ∆*dhaD*∆*ldhA,* increasing to 80 % in *C. werkmanii* ∆*dhaD*∆*adhE*. The final 3-HPA concentration in the latter is far below the critical concentration [5]. These positive effects are most likely due to the rebalance of the NAD^+^/NADH ratio (Table 2). The ratio was decreased from 4.74 ± 0.14 for *C. werkmanii* ∆*dhaD* to 1.66 ± 0.03 and 2.20 ± 0.07 for *C. werkmanii* ∆*dhaD*∆*ldhA* and *C. werkmanii* ∆*dhaD*∆*adhE*, respectively, which is much closer to the NAD^+^/NADH ratio of the wild-type *C. werkmanii* (1.58 ± 0.25).

The other metabolic fluxes are redistributed as well in the double knock-outs (Table 3). Comparing *C. werkmanii* ∆*dhaD* with *C. werkmanii* ∆*dhaD*∆*ldhA*, the ethanol concentration decreases, while the acetate concentration increases. This indicates that the flux from acetyl-CoA to acetate is increased, while the flux from acetyl-CoA to ethanol is reduced. Analogously, an enhanced acetate titer can be observed in *C. werkmanii* ∆*dhaD*∆*adhE*. Due to the *adhE* deletion, four NADH molecules become available per glucose, which are used in other NADH-consuming reactions. As a result, compared to the single ∆*dhaD* mutant, not only the PDO titer is elevated, but also the concentration of succinate and especially lactate, analogous to the single *adhE* knock-out compared to the wild-type.

In the triple knock-out mutant, the lactate concentration decreases till below the detection limit and the succinate titer stagnates. Moreover, the final PDO titer and yield on glycerol are further improved. The yield even reaches the maximum theoretical yield of 1.00 ± 0.03 mol PDO/mol glycerol with the use of a co-substrate. As such, at the end of this experiment, 3-HPA is no longer detected. The residual glycerol and glucose concentrations are increased in the triple knock-out mutant, compared to the single knock-out strain. The growth rate is reduced and the final pH is increased (Table 2), probably due to a metabolic burden.

### Performance of *C. werkmanii ∆dhaD∆ldhA∆adhE::*ChlFRT in bioreactors

The triple mutant *C. werkmanii* ∆*dhaD*∆*ldhA*∆a*dhE*::ChlFRT producing the maximum theoretical yield of 1 mol PDO/mol glycerol in minimal medium with glycerol and glucose in shake flasks, yielding a titer of 107.20 ± 4.22 mM PDO, was selected for batch fermentations on bioreactor scale. As the wild-type *C. werkmanii* DSM17579 showed the highest productivity at an initial concentration of 650 mM glycerol [18], this concentration was used for the batch fermentations on bioreactor scale with the triple mutant. Analogous to the shake flask experiments, a ratio of 0.33 mol glucose/mol glycerol was used.

**Fig. 1:**
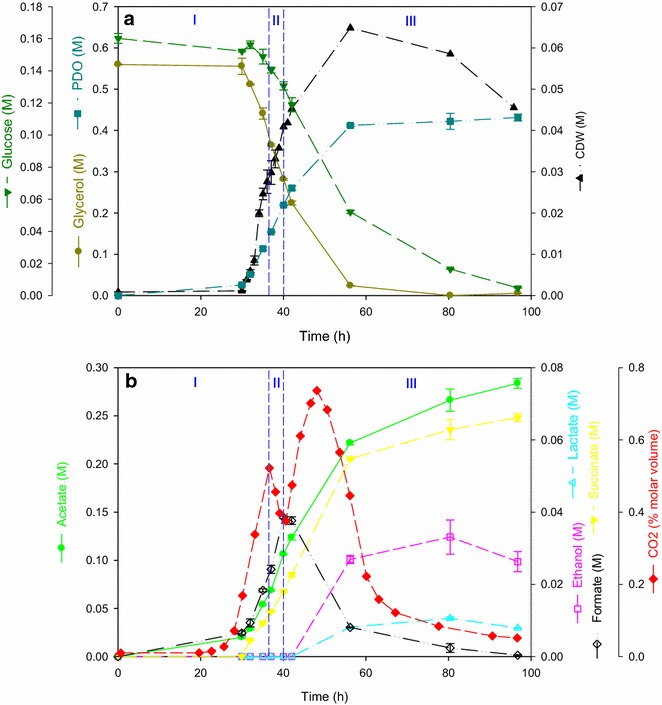
Profile of the batch fermentation on bioreactor scale with *C. werkmanii* ∆*dhaD*∆*ldhA*∆*adhE*::ChlFRT. The strain was grown in fermentation medium with 220 mM glucose and 650 mM glycerol under anaerobic conditions. The cell dry weight is estimated from OD_600nm_ measurements as described in the “Methods” section. The symbols in graph **a** represent () CDW, () glycerol, () glucose, and () PDO; in graph **b** () acetate, () ethanol, () lactate, () succinate, () formate, and () CO_2_

The maximum growth rate of the triple knock-out mutant obtained in the bioreactor is 0.11 ± 0.01 h^−1^ with a lag phase of around 30 h. During the fermentation on bioreactor scale, glycerol and glucose are consumed simultaneously (Fig. 1a), but the consumption rate of glycerol is 10 times higher than that of glucose (29.10 ± 1.10 mmol glycerol/h and 3.70 ± 0.30 mmol glucose/h). As a result, glycerol is depleted first, namely at the end of the exponential phase. The residual glucose is not used anymore for growth, but only for cell maintenance and production of acids and ethanol (Fig. 1b). These findings indicate that a reduced molar ratio glucose/glycerol may be desirable on bioreactor scale. The PDO productivity during the growth, i.e. production phase, 14.07 ± 0.65 mM PDO/h, is in line with the results obtained with the wild-type strain in fermentation medium with glycerol as sole carbon source, during the same phase [18]. This indicates a recovery of the cofactor balance, which was lopsided in *C. werkmanii* ∆*dhaD.*

The final titer of PDO is increased by 32 % comparing the cultivation of the triple knock-out (422.01 ± 19.37 mM) with that of the wild-type strain in fermentation medium with glycerol as sole carbon source. To further improve this titer, several strategies may be followed. The reactor mode can be switched from batch fermentation to fed-batch or continuous fermentation. The NADH-dependent 1,3-propanediol dehydrogenase can be replaced by an NADPH-dependent oxidoreductase. Several researchers have used the oxidoreductase of *E. coli* to enhance the production of PDO successfully in *E. coli* and *Klebsiella* species [19–21]. Lastly, a more holistic approach can be applied, such as adaptive evolution [22] and global transcriptional machinery engineering (gTME) [23].

While PDO is the main product, some byproducts are detected (Fig. 1b). The most important one is acetate (266.19 ± 11.50 mM), mainly produced during the exponential growth phase, and with a production profile similar to the one of glucose consumption. Succinate follows the same production profile, but to a much lower final titer (62.81 ± 2.76 mM). In the first half of the exponential growth phase, no lactate or ethanol is produced, but by the end of the exponential phase (period III) the triple knock-out does produce lactate and ethanol, albeit in minimal concentrations. It should be noted that the produced lactate may be L-lactate instead of D-lactate, since these metabolites cannot be separated with the used HPLC-method. Analogous results were obtained in a fed-batch fermentation on bioreactor scale with an *ldhA* deficient *K. pneumoniae* strain, i.e. lactate started accumulating in the late-exponential phase [9]. Ethanol may be produced by an iron-containing alcohol dehydrogenase, EutG, which is present in *Citrobacter* sp. next to AdhE [24, 25]. A peculiar production profile is found for formate, which is produced early exponential, but consumed from the mid-exponential phase. According to Beauprez [13], this can be ascribed to the activity of the formate hydrogen lyase (FHL, E.C. 1.2.1.2), which converts formate and NAD^+^ in CO_2_ and NADH. When enough NADH molecules are present in the cell, FHL is not active and formate accumulates. However, during shortage or surplus of NADH, the enzyme is active. In the former case, formate will be converted to CO_2_ and NADH, while in the latter case, formate and NAD^+^ will be produced [13]. Based on the above observations, the experiment can be divided in three phases: (I) from 0 h to 36.5 h; (II) from 36.5 h to 40 h; and (III) from 40 h till the end of the fermentation. In period I enough NADH is present for the cell and formate accumulates. In period II, a surplus of NADH exists and CO_2_ is converted to formate. This is reflected in a drop of the CO_2_-curve and further increase of the formate concentration. Finally, in period III, the activity of FHL is reversed, formate is converted to CO_2_ and NADH resulting in a lift of the CO_2_ concentration and reduced formate concentration. The produced NADH is consumed by other enzymes, such as dehydrogenases, to form ethanol and lactate.

All glycerol and glucose is depleted by the end of the batch cultivation on bioreactor scale, but the PDO yield on glycerol is lower than expected, namely 0.73 ± 0.01 mol PDO/mol glycerol. Nevertheless, this is still one of the highest reported PDO yields on glycerol [11]. The closest results, 0.70 and 0.69 mol PDO/mol glycerol, were accomplished by, respectively, Zhang et al. [10] using the knock-out *K. pneumoniae* YMU2 ∆*aldA* in fed-batch mode and Seyfried et al. [26] with the thermophilic wild-type *Caloramator viterbensis* sp. nov. in Hungate tubes. Since the only intermediate in the conversion of glycerol to PDO is 3-HPA and neither 3-HPA was accumulated nor unknown peaks were visible in the HPLC chromatographs, glycerol must have been used for growth and maintenance of the cell. This hypothesis is strengthened by the calculated carbon and redox balances (Additional file 1: Table S1). When the conversion of glycerol to PDO is taken into account, 28.52 % of the carbon has ‘disappeared’, while when the conversion of glucose to the biomass and byproducts is considered, a surplus of 90.27 % carbon would be needed. However, when both pathways are considered together, a carbon and redox balance of, respectively, 95.56 % and 92.25 % are obtained, which confirms that glycerol is used not only for production of PDO, but also for cell growth (and maintenance).

In order to confirm this hypothesis, a bioreactor with only 650 mM glycerol, thus without glucose, was inoculated with the triple knock-out strain. After a lag phase of 70 h, the strain started to grow, consumed glycerol and produced PDO (Table 4). So, although GDH, coded by *dhaD* and responsible for channeling glycerol to the central metabolism, is altered by gene deletion in this triple knock-out, the strain still uses glycerol as carbon source for cell growth and maintenance, indicating the presence of other enzymes which convert glycerol into central carbon metabolites supporting growth. The maximal cell dry weight of the triple mutant grown on glycerol only is 38.90 ± 2.07 mM, which is lower than on glucose and glycerol. This is as expected because in the former, glycerol is the only carbon source for cell growth and maintenance, while in the latter, glucose is also present in the medium. In both cases, the 3-HPA concentration is below the detection limit and the PDO yield on glycerol and the final PDO titer obtained are similar (Table 4). Furthermore, the metabolic profile is similar for both conditions, taking the difference in carbon concentration into account. The most important byproduct still is acetate and small amounts of succinate and lactate are formed. However, the production of ethanol is not detected anymore. In contrast, the residual glycerol concentration differs in both cases. The glycerol concentration is depleted when glycerol and glucose are used together as carbon sources, whereas 93.14 ± 1.97 mM glycerol is still present in the other condition. Moreover, the glycerol uptake rate is higher when both substrates are used (data not shown).

**Table 4:** Comparison of batch cultivations [glycerol (Gly), or glycerol and glucose (Glu + Gly)] with *C. werkmanii* ∆*dhaD*∆*ldhA*∆*adhE::*ChlFRT

Strain	Yield (mol PDO/mol glycerol)	Residual concentration (mM)		Metabolite concentration (mM)					CDW
		Glycerol	Glucose	PDO	Acetate	Ethanol	Lactate	Succinate	
Gly	0.72 ± 0.05	93.14 ± 1.97	ND	404.80 ± 8.51	166.76 ± 3.49	BDL	5.86 ± 0.01	25.74 ± 0.44	38.83 ± 2.07
Glu + Gly	0.73 ± 0.01	BDL	16.51 ± 0.03	422.01 ± 19.37	266.19 ± 11.50	33.11 ± 4.74	10.60 ± 0.38	62.81 ± 2.76	64.82 ± 2.84

The strain was grown on bioreactor scale in fermentation medium under anaerobic conditions. The cell dry weight is estimated from OD_600nm_ measurements as described in “Methods” section. The values are the average of two experiments with their corresponding standard deviations

*ND* not determined, *BDL* below detection limit

These results give rise to the question which enzyme is responsible for the conversion of glycerol to biomass and byproducts. According to literature, the first enzyme of the oxidative pathway in PDO-producing microorganisms under anaerobic conditions is glycerol dehydrogenase, GDH, which converts glycerol to dihydroxyacetone [2]. The function of this enzyme has been confirmed for *C. werkmanii* DSM17579 [5]. Thus, the triple knock-out, lacking GDH, is not expected to grow on glycerol anaerobically. A first candidate enzyme which could explain the growth is glycerol kinase (GK, E.C. 2.7.1.30), which converts glycerol to *sn*-glycerol-3-phosphate using ATP. Although this enzyme is normally only active in the presence of an exogenous electron acceptor, it might have been activated to replace GDH. A second possibility is that a promiscuous dehydrogenase enzyme has taken over the task of GDH and converts glycerol to DHA. To check these theories, enzyme assays were performed using crude cell extract of the two bioreactor cultivations to check the activity of GDH and GK (Table 5). The specific activity of GK is very low and almost no difference is observed between the cells grown on glycerol alone and those grown on glycerol and glucose. Therefore, it is not likely that a GK enzyme is responsible for the dissimilation of glycerol via the oxidative pathway. The activity of GDH on the other side is almost double the glycerol kinase activity (Table 5). Furthermore, when the triple mutant was grown under anaerobic conditions in medium with glycerol as sole carbon source, the specific GDH activity is three times higher than when the strain is grown under the same conditions in medium with glycerol and glucose. These results indicate that a promiscuous dehydrogenase is responsible for the utilization of glycerol for cell growth and maintenance, or that a second glycerol dehydrogenase coding gene is present in the genome of *C. werkmanii* DSM17579.

**Table 5:** The glycerol dehydrogenase and glycerol kinase activity (mU/mg protein) of *C. werkmanii* ∆*dhaD*∆*ldhA*∆*adhE::ChlFRT*

Carbon source	Enzyme activity (mU/mg protein)	
	Glycerol dehydrogenase	Glycerol kinase
Glycerol	72.70 ± 1.76	10.67 ± 1.34
Glycerol + Glucose	26.80 ± 0.18	13.85 ± 0.20

The strain was grown under anaerobic conditions in fermentation medium with glycerol only or with 0.33 molar ratio glucose to glycerol in batch cultivations on bioreactor scale. The values are the averages of two experiments with their corresponding standard deviations

**Fig. 2:**
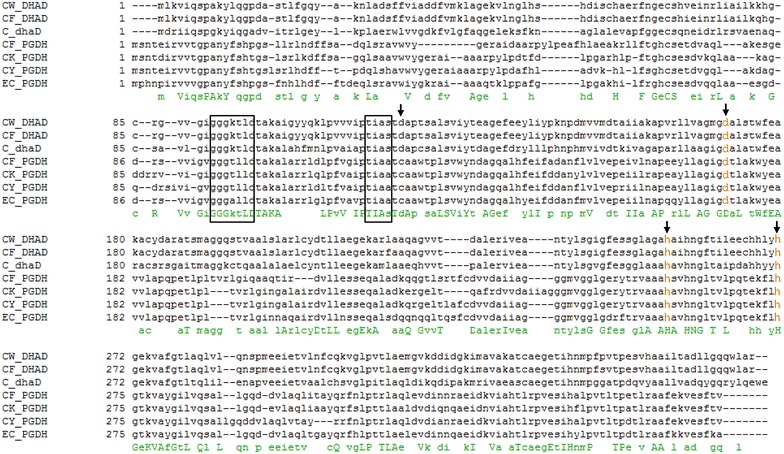
Amino acid homologies between putative glycerol dehydrogenases (PGDH) and known glycerol dehydrogenases (DHAD) coded by *dhaD*. The amino acids of CF_DHAD (*Citrobacter freundii,* GenBank: EHL83381.1), CW_DHAD (*C. werkmanii* DSM17579, GenBank: AFX65883.1), C_PGDH (*Citrobacter* sp., GenBank: WP_042998939.1), CY_PGDH (*C. youngae* ATCC29220, GenBank: EFE08361.1), CK_PGDH (*C. koseri* ATCC BAA-895, GenBank: ABV13669.1), and EC_PGDH (*Escherichia coli* K12 MG1655, GenBank: NP_415132.1) are aligned. Matching amino acids are marked under the alignment. The putative NAD-binding sites are *boxed*, the putative glycerol binding sites are marked with an *arrow*, and the putative manganese binding sites are *orange*

An NCBI-BLAST of the *C. werkmanii* DSM17579 glycerol dehydrogenase to the protein sequences of *Citrobacter* species indeed reveals a second, putative glycerol dehydrogenase enzyme [27]. This putative glycerol dehydrogenase shows 32 % amino acid identity to the glycerol dehydrogenase enzyme coded by *dhaD* of *Citrobacter* sp. (GenBank: WP_042998939.1, E-value = 1e^−39^, bitscore = 147). In Fig. 2 a comparison is made between the amino acid sequences of GDH’s coded by *dhaD* of *Citrobacter* sp. (GenBank: WP_042998939.1) *C. werkmanii* DSM17579 (GenBank: AFX65883.1) and *C. freundii* (GenBank: AAB48844.1), and putative glycerol dehydrogenases found in *Citrobacter* sp. (GenBank: ABV13669.1, EFE08361.1, EHL83381.1) and *E. coli* (GenBank: NP_41532.1). Glycerol dehydrogenases belong to the family of iron-dependent alcohol dehydrogenases [28]. Surprisingly, the conserved regions of GDH’s, as described in Maervoet et al. [29], can all be found in this putative glycerol dehydrogenase. The NAD^+^- (marked as boxes) and Manganese- (orange) binding sites are well conserved suggesting that these two elements also are the cofactors for this second, putative enzyme, as they are for the GDH coded by *dhaD*. Even the glycerol binding sites (marked with an arrow) are conserved, except for Asp121 in GDH coded by *dhaD*, which was replaced by Cys123 in the putative glycerol dehydrogenase. As such, we presume that this putative glycerol dehydrogenase replaces the GDH activity coded by *dhaD* in the triple knock-out mutant and that this enzyme is responsible for the cell growth and maintenance. However, a knock-out mutant of this putative glycerol dehydrogenase gene should be created to confirm the hypothesis.

Taken together, the most promising process is the one where glycerol and glucose are both used as co-substrates, as the productivity is the highest (4.35 ± 0.20 mM/h for both substrates and 3.15 ± 0.07 mM/h for glycerol only), and the residual glycerol concentration is below the detection limit.

## Conclusions

In this study, genes were deleted coding for non-essential NADH-consuming enzymes to examine the reduction of the 3-HPA concentration and the related enhancement of the final PDO titer and yield on glycerol. The triple mutant *C. werkmanii* ∆*dhaD*∆*ldhA*∆*adhE* reached the maximum theoretical yield of 1.00 ± 0.03 mol PDO/mol glycerol, and a final titer of 107.20 ± 4.22 mM PDO when grown in shake flasks on glucose and glycerol as carbon sources under anaerobic conditions. When the strain was grown on glycerol and glucose on bioreactor scale, glycerol was depleted, the toxic intermediate 3-HPA was below the detection limit and 422.01 ± 19.37 mM PDO was produced.

## Methods

All chemicals were obtained from Sigma-Aldrich (Belgium), unless otherwise stated.

### Strains and plasmids

The strains used in this work are summarized in Table 6. The different strains were preserved in a (1:1) glycerol (70 % v/v):LB (Luria Broth)-medium solution. The plasmids used are described in Maervoet et al. [5].

**Table 6:** Bacterial strains used in this work

Strains	Reference
*C. werkmanii* DSM17579	DSMZ, Braunschweig, Germany
*C. werkmanii* DSM17579 ∆*dhaD*	Maervoet et al. [5]
*C. werkmanii* DSM17579 ∆*ldhA*	This study
*C. werkmanii* DSM17579 ∆*adhE*::ChlFRT	This study
*C. werkmanii* DSM17579 ∆*arcA*::ChlFRT	This study
*C. werkmanii* DSM17579 ∆*dhaD*∆*ldhA*	This study
*C. werkmanii* DSM17579 ∆*dhaD*∆*adhE*::ChlFRT	This study
*C. werkmanii* DSM17579 ∆*dhaD*∆*ldhA*∆*adhE*::ChlFRT	This study

*ChlFRT* chloramphenicol cassette flanked by FRT sites

### Genetic methods

The primers (Additional file 1: Table S2) to unravel a part of or the complete sequence of *adhE*, *ldhA* and *arcA* of *C. werkmanii* DSM17579 were developed by comparing the homologous regions of the genes from *C. koseri* ATCC BAA-895, *C. rodentium* ICC168, and *Citrobacter* sp. 30_2. The genes were picked up by PCR with Taq DNA polymerase (New England Biolabs, Belgium), cloned in the pGEM-T vector (Promega, Belgium), and transformed in chemically competent *E. coli* DH5α cells [30]. The genes were sequenced (LGC Genomics, Germany) using the primers SP6bis and T7bis (Additional file 1: Table S2). The sequence data of *ldhA* and *arcA* were submitted to the Genbank database and assigned accession number KJ957079 and KJ957080, respectively.

The primers used for the knock-out protocol were developed using the unraveled gene sequences, as summarized in (Additional file 1:Table S2). The method to create the knock-out mutants is described in Maervoet et al. [5] and in Additional file 3. All knock-out mutants were confirmed by colony PCR and sequencing using the control primers. The sequences of the knock-out strains are presented in Additional file 2. When multiple genes were knocked-out in *C. werkmanii* DSM17579, all deletions were double checked to see whether no other chromosomal rearrangements had occurred due to the flippase recognition target scars. The chloramphenicol resistance cassette was not removed from the knock-out strains *C. werkmanii* ∆*adhE*::ChlFRT, *C. werkmanii* ∆*dhaD*∆*adhE*::ChlFRT, and *C. werkmanii* ∆*dhaD*∆*ldhA*∆*adhE*::ChlFRT.

### Media and cultivation conditions

The cultivation medium [163 mM glycerol as sole carbon source or 40 mM glucose and 120 mM glycerol (0.33 mol/mol glucose/glycerol)] and conditions as described in Maervoet et al. [18] were used for the shake flask experiments.

The growth medium and cultivation conditions for the reactor experiments are described in Maervoet et al. [18]. 220 mM glucose and 650 mM glycerol were used as C-source. The CO_2_ was measured with an EL3020 off-gas analyzer (ABB Automation GnbH, Germany) and the data were logged with the Sartorius MFCS/win v3.0 system (Sartorius Stedim Biotech, Germany).

### Analytical methods

The biomass concentration was measured as absorbance at 600 nm. During the fermentation experiments, the value of the optical density was converted to cell dry weight by an appropriate calibration curve. A molecular weight of 25.73 g/mol was used to convert the cell dry weight from g/L to M. Glycerol, PDO, lactate, acetate, succinate, formate and ethanol were quantified with an HPLC system (Varian, Belgium) coupled with a Refractive Index Detector and a dual UV Detector (wavelength of 210 and 265 nm). The compounds were separated by using an Aminex 300 × 7.8 mm HPX-87H Organic Acid Analysis Column (Bio-Rad Laboratories, Belgium) and eluted at 600 µL/min isocratically in 5 mM H_2_SO_4_ at 65 °C. Glucose was analyzed using the YSI 2700 SELECT Biochemistry Analyzer (YSI Life Sciences, Ankersmid Scientific, Belgium). 3-hydroxypropionaldehyde (3-HPA) was determined by an HPLC system with a Rezex ROA Organic Acid Analysis column (Phenomenex, Belgium) using a dual Ultraviolet Detector with a wavelength of 210 and 265 nm. The metabolite was eluted at 500 µL/min isocratically in 10 mM H_2_SO_4_ at 40 °C.

### Determination of enzyme activities

The preparation of the cell free extract and the enzyme test for glycerol dehydrogenase are described in Maervoet et al. [5].

The assay mixture to determine the glycerol kinase activity, with a total volume of 3 mL, contained 0.7 mL reagent solution, 0.28 M glycine with 30 mM potassium carbonate (pH 8.9), and 0.033 M glycerol. The reagent solution contained 8.5 mM ATP, 1.22 mM NADH, 2 mM phosphoenol pyruvate, 15.3 U/mL lactate dehydrogenase, 7 U/mL pyruvate kinase, 28 mM MgSO_4_.7H_2_O, and 26 mM reduced glutathione (pH 7.4). The reaction was started by the addition of crude cell extract diluted in 0.1 M triethanolamine buffer (pH 7.4) to the assay mixture. The reaction velocity was measured in a coupled system with pyruvate kinase and lactate dehydrogenase. One unit is defined as the oxidation of 1.0 µmol of NADH per min at 25 °C and pH 8.9. Protein concentrations were measured using the BCA Protein Assay Kit from Thermo Scientific (Belgium).

### Quantification of NADH and NAD^+^ concentrations

NADH and NAD^+^ concentrations were determined using Enzychrom NAD^+^/NADH assay kit (Gentaur, Belgium) following the manufacturer’s protocol. The assay utilizes alcohol dehydrogenase for NAD(H) quantification. Colorimetric changes in the samples were measured at 565 nm.

## Additional files

10.1186/s12934-016-0421-ySimplified scheme of glycerol metabolizing pathways and rational engineering strategy. **Figure S2.** A comparison of the carbon metabolism in E. coli under (A) aerobic and (B) anaerobic conditions [12]. **Table S1.** Carbon and redox balances. **Table S2.** Primers used in the study. 
10.1186/s12934-016-0421-y Sequence data confirming different knock-out strains. Green = sequence of P1 primer; blue = sequence of P2 primer; red = FRT scar; purple = chloramphenicol resistance gene.
10.1186/s12934-016-0421-y Detailed, optimized protocol for the creation of a knock-out in *Citrobacter werkmanii* DSM17579.

## Abbreviations

3-HPA: 3-hydroxypropionaldehyde
FHL: formate hydrogen lyase
GDH: glycerol dehydrogenase
GK: glycerol kinase
PDO: 1,3-propanediol
PDODH: 1,3-propanediol dehydrogenase
