# Supplementary material for: High yield 1,3-propanediol production by rational engineering of the 3-hydroxypropionaldehyde bottleneck in Citrobacter werkmanii
Source: Microb Cell Fact. 2016 Jan 28;15:23. doi: 10.1186/s12934-016-0421-y (PMC4731958; doi:10.1186/s12934-016-0421-y)
Supplement: Supplementary file 1 — 10.1186/s12934-016-0421-ySimplified scheme of glycerol metabolizing pathways and rational engineering strategy. Figure S2. A comparison of the carbon metabolism in E. coli under (A) aerobic and (B) anaerobic conditions [12]. Table S1. Carbon and redox balances. Table S2. Primers used in the study. [file 12934_2016_421_MOESM1_ESM.pdf]

Additional file 1

High yield 1,3-propanediol production by rational engineering of the 3-hydroxypropionaldehyde bottleneck in Citrobacter werkmanii

Veerle ET Maervoet[1], Sofie L De Maeseneire, Fatma G Avci[2], Joeri Beauprez, Wim K Soetaert and Marjan De Mey[*]

Centre of Expertise - Industrial Biotechnology and Biocatalysis, Department of Biochemical and Microbial Technology, Ghent University, Coupure links 653, B-9000 Ghent, Belgium

[1]Present address: Laboratory of Biochemistry and Brewing, Department of Applied Bioscience Engineering, Ghent University, Valentin Vaerwyckweg 1, 9000 Ghent

[2]Present address: Bioengineering Department, Faculty of Engineering, Ege University, 35100 Bornova-Izmir, Turkey

[*]Corresponding author:

Marjan De Mey

Phone: +32 9 264 60 28

Fax: +32 9 264 62 48

e-mail: Marjan.DeMey@UGent.be

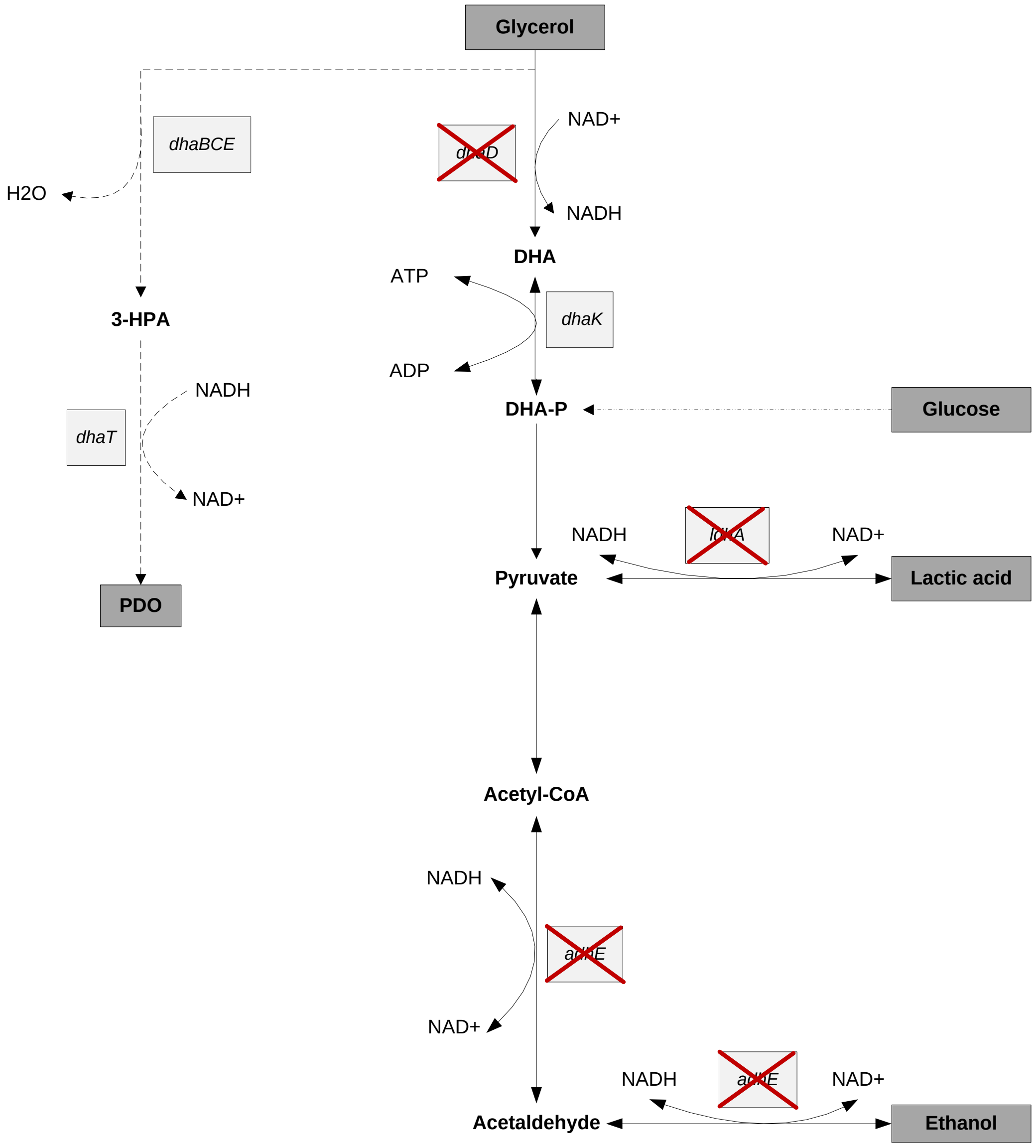

Figure S1: Simplified scheme of glycerol metabolizing pathways and rational engineering strategy. The dashed lines are reactions of the reductive pathway, the solid lines represent the oxidative pathway and the dotted line the entrance of the co-substrate glucose necessary when *dhaD* is knocked out. The different genes which are knocked out during the experiments are crossed out. Only the relevant genes and metabolites are drawn. PDO = 1,3-propanediol; DHA = dihydroxyacetone; DHA-P = dihydroxyacetone phosphate; *dhaBCE* = glycerol dehydratase; *dhaT* = 1,3-propanediol dehydrogenase; *dhaD* = glycerol dehydrogenase; *dhaK* = dihydroxyacetone kinase; *ldhA* = lactate dehydrogenase; *adhE* = ethanol dehydrogenase

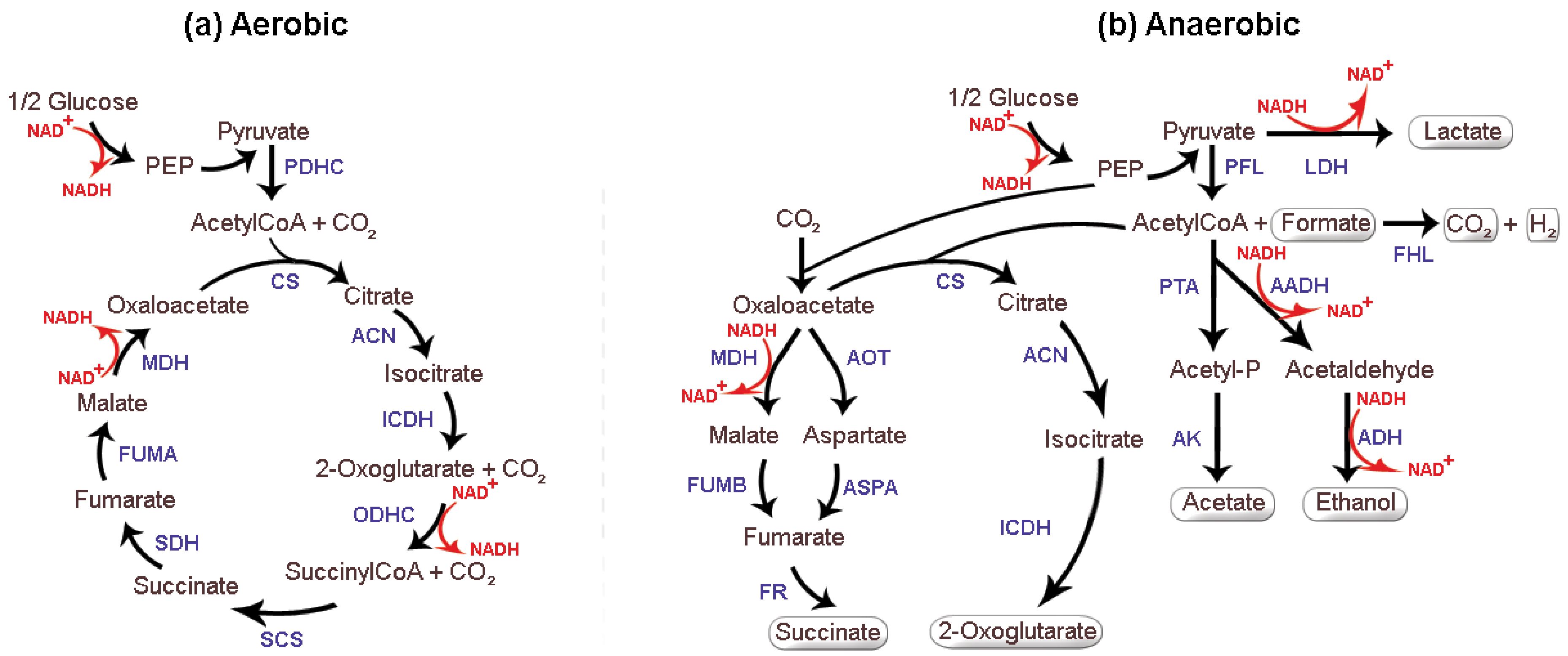

Figure S2: A comparison of the carbon metabolism in *E. coli* under (A) aerobic and (B) anaerobic conditions. PDHC = pyruvate:ferredoxin oxidoreductase, CS = citrate synthase, ACN = aconitase, ICDH = isocitrate dehydrogenase, ODHC = 2-oxoglutarate dehydrogenase, SCS = succinate thiokinase, SDH = succinate dehydrogenase, FUMA, FUMB = fumarase, MDH = malate dehydrogenase, PFL = pyruvate-formate lyase, FR = fumarate reductase, AOT = aspartate aminotransferase, ASPA = aspartate ammonia-lyase, LDH = D-lactate dehydrogenase, PTA = phosphotransacetylase, AK = acetate kinase, AADH = acetaldehyde dehydrogenase, ADH = alcohol dehydrogenase, FHL = formate-hydrogen lyase (after White, 1995).

Table S1: **Carbon and redox balances.** Calculations for the different pathways during the batch fermentations on bioreactor scale with *C. werkmanii* DSM17579 Δ*dhaD*Δ*ldhA*Δ*adhE*::ChlFRT in fermentation medium with 220 mM glucose and 650 mM glycerol under anaerobic conditions.

**Reaction**	**Carbon (%)**	**Redox (%)**
Glycerol to PDO	71.48±1.44	81.69±1.44
Glucose to biomass and byproducts	190.27±4.92	140.72±5.08
Glycerol and glucose to PDO, biomass, and byproducts	95.56±11.17	92.25±9.96

Table S2: Primers used in the study. Primers to unravel the sequence of the *ldhA, adhE,* and *arcA* genes and to create (P1 and P2) and verify (out) the different single and multiple knock-out strains.

**Primername**	**Sequence (5'→ 3')**
Picking up genes	
ldhACitro-Fw	CCGAGGAATTCGGAGGAAACAAAGATGAAACTCGCCGTCTACAGCACCAAACAG
ldhACitro-Rv	CGCTATACGCGTTAAAACAGGGCGTTTGGACAGCTTTC
Fw-*arcA*	ATGCAGACCCCGCACATTCTTATCGTTGAAGACGAGTTGG
Rv-*arcA*	TTAGTCCTGTAGATCACCGCAGAAACGGTAACCTTCACCGTGAATGG
Fw-*adhE*3	TTGCTGAGCTGGCAGGCTTCTCCGTACCAGAAACC
Rv-*adhE*3	CCGTAGTAGGTATCCAGCAGAATCTGTTTCAGCTCGGAGATCAGCG
SP6bis	CGCCAAGCTATTTAGGTGAC
T7bis	GGCGATTAAGTTGGGTAACG
Linear DNA (P1 and P2)	
Fw-*ldhA*-P1	CAGCACCAAACAGTACGACAAAAAGTATCTGCAGCAGGTTAACGAGGCTTTTGGGTGTAGGCTGGAGCTGCTTC
Rv-*ldhA*-P2	TTGTTTCAGATTTTGCAGAGTCGTTTCAGAAATGCTGGTCAACGCTTCGGCGCATATGAATATCCTCCTTAG
ldhA-H1'-bis	ATGAAACTCGCCGTCTACAGCACCAAACAGTACGACAAAAAG
ldhA-H2'-bis	CAGGGCGTTTGGACAGCTTTCGCCTTTGGCGATTTGTTTCAGATTTTGCAGAGTCGTTTC
Fw-*arcA*-P1	ATGCAGACCCCGCACATTCTTATCGTTGAAGACGAGTTGGTAACACGCAACACGTTGAAAAGCATTTTCGGTGTAGGCTGGAGCTGCTTC
Rv-*arcA*-P2	TTAGTCCTGTAGATCACCGCAGAAACGGTAACCTTCACCGTGAATGGTAGCGATGATTTCTGGCGTATCCGGCGTTGATTCGAAATGTTTACGAATGCGGCATATGAATATCCTCCTTAG
Fw-*adhE*-P1	GCGGGTTAGCACCAGTACACTGGTCGTCAAACGCATCTTCAGACAGTTTGTCAACGTGTGCCAGGAAGTCAGCTTCCTGCACGCCAGCTTCACGGATAGGTGTAGGCTGGAGCTGCTTC
Rv-*adhE*-P2	ATGGCTGTTACTAATGTCGCTGAACTTAACGCACTTGTAGAGCGCGTAAAAAAAGCCCAGCGTGAATATGCCAGTTTCACTCAAGAACAGGTTGACCATATGAATATCCTCCTTAG
Control primers (out)	
ldhA-out-Fw	GTATCTGCAGCAGGTTAACG
ldhA-out-Rv	CGTTTCAGAAATGCTGGTCAACG
arcA-out-Fw	GAGTTGGTAACACGCAACAC
arcA-out-Rv	CGATGATTTCTGGCGTATCC
adhE-out-Fw	AACGTGTGCCAGGAAGTCAG
adhE-out-Rv	AAAGCCCAGCGTGAATATGC
