# Supplementary material for: High yield 1,3-propanediol production by rational engineering of the 3-hydroxypropionaldehyde bottleneck in Citrobacter werkmanii
Source: Microb Cell Fact. 2016 Jan 28;15:23. doi: 10.1186/s12934-016-0421-y (PMC4731958; doi:10.1186/s12934-016-0421-y)
Supplement: Supplementary file 2 — 10.1186/s12934-016-0421-y Sequence data confirming different knock-out strains. Green = sequence of P1 primer; blue = sequence of P2 primer; red = FRT scar; purple = chloramphenicol resistance gene. [file 12934_2016_421_MOESM2_ESM.pdf]

Additional file 2

High yield 1,3-propanediol production by rational engineering of the 3-hydroxypropionaldehyde bottleneck in Citrobacter werkmanii

Veerle ET Maervoet[1], Sofie L De Maeseneire, Fatma G Avci[2], Joeri Beauprez, Wim K Soetaert and Marjan De Mey[*]

Centre of Expertise - Industrial Biotechnology and Biocatalysis, Department of Biochemical and Microbial Technology, Ghent University, Coupure links 653, B-9000 Ghent, Belgium

[1]Present address: Laboratory of Biochemistry and Brewing, Department of Applied Bioscience Engineering, Ghent University, Valentin Vaerwyckweg 1, 9000 Ghent

[2]Present address: Bioengineering Department, Faculty of Engineering, Ege University, 35100 Bornova-Izmir, Turkey

[*]Corresponding author:

Marjan De Mey

Phone: +32 9 264 60 28

Fax: +32 9 264 62 48

e-mail: Marjan.DeMey@UGent.be

Sequence data confirming different knock-out strains. Green = sequence of P1 primer; blue = sequence of P2 primer; red = FRT scar; purple = chloramphenicol resistance gene

dhaD: 5’-
GCATAGCCATGACATTAGCTGCCACGCGGGTGTAGGCTGGAGCTGCTTCGAAGTTCCTATACTTTCTAGAGAA
TAGGAACTTCGGAATAGGAACTAAGGAGGATATTCATATGGCGGAAATGGGCGTAAAAGATGACATTGATGG
CAAGATCATGGCCGTG-3’

ldhA: 5’-
TCTGCAGCAGGTTAACGAGGCTTTTGGGTGTAGGCTGGAGCTGCTTCGAAGTTCCTATACTTTCTAGAGAATA
GGAACTTCGGAATAGGAACTAAGGAGGATATTCATATGCGCCGAAGCGTTGACCAGCATTTCTGAAACG-3’

adhE::ChlFRT: 5’-
GGTGTAGGCTGGAGCTGCTTCGAAGTTCCTATACTTTCTAGAGAATAGGAACTTCGGAATAGGAACTTCATTTA
AATGGCGCGCCTTACGCCCCGCCCTGCCACTCATCGCAGTACTGTTGTAATTCATTAAGCATTCTGCCGACATG
GAAGCCATCACAAACGGCATGATGAACCTGAATCGCCAGCGGCATCAGCACCTTGTCGCCTTGCGTATAATAT
TTGCCCATGGTGAAAACGGGGGCGAAGAAGTTGTCCATATTGGCCACGTTTAAATCAAAACTGGTGAAACTCA
CCCAGGGATTGGCTGAGACGAAAAACATATTCTCAATAAACCCTTTAGGGAAATAGGCCAGGTTTTCACCGTA
ACACGCCACATCTTGCGAATATATGTGTAGAAACTGCCGGAAATCGTCGTGGTATTCACTCCAGAGCGATGAA
AACGTTTCAGTTTGCTCATGGAAAACGGTGTAACAAGGGTGAACACTATCCCATATCACCAGCTCACCGTCTTT
CATTGCCATACGTAATTCCGGATGAGCATTCATCAGGCGGGCAAGAATGTGAATAAAGGCCGGATAAAACTTG
TGCTTATTTTTCTTTACGGTCTTTAAAAAGGCCGTAATATCCAGCTGAACGGTCTGGTTATAGGTACATTGAGCA
ACTGACTGAAATGCCTCAAAATGTTCTTTACGATGCCATTGGGATATATCAACGGTGGTATATCCAGTGATTTTT
TTCTCCATTTTAGCTTCCTTAGCTCCTGAAAATCTCGACAACTCAAAAAATACGCCCGGTAGTGATCTTATTTCAT
TATGGTGAAAGTTGGAACCTCTTACGTGCCGATCAACGTCTCATTTTCGCCAAAAGTTGGCCCAGGCTTCCCGG
TATCAACAGGGACACCAGGATTTATTTATTCTGCGAAGTGATCTTCCGTCACAGGTAGGCGCGCCGAAGTTCCT
ATACTTTCTAGAGAATAGGAACTTCGGAATAGGAACTAAGGAGGATATTCATA-3’

arcA: 5’-
TTGGTAACACGCAACACGTTGAAAAGCATTTTCGGTGTAGGCTGGAGCTGCTTCGAAGTTCCTATACTTTCTAG
AGAATAGGAACTTCGGAATAGGAACTAAGGAGGATATTCATATGCCGCATTCGTAAACATTTCGAATCAACGC
CGGATACGCCAGAAATCATCGA-3’
