# Supplementary material for: High yield 1,3-propanediol production by rational engineering of the 3-hydroxypropionaldehyde bottleneck in Citrobacter werkmanii
Source: Microb Cell Fact. 2016 Jan 28;15:23. doi: 10.1186/s12934-016-0421-y (PMC4731958; doi:10.1186/s12934-016-0421-y)
Supplement: Supplementary file 3 — 10.1186/s12934-016-0421-y Detailed, optimized protocol for the creation of a knock-out in Citrobacter werkmanii DSM17579. [file 12934_2016_421_MOESM3_ESM.pdf]

Additional file 3

High yield 1,3-propanediol production by rational engineering of the 3-hydroxypropionaldehyde bottleneck in Citrobacter werkmanii

Veerle ET Maervoet[1], Sofie L De Maeseneire, Fatma G Avci[2], Joeri Beauprez, Wim K Soetaert and Marjan De Mey[*]

Centre of Expertise - Industrial Biotechnology and Biocatalysis, Department of Biochemical and Microbial Technology, Ghent University, Coupure links 653, B-9000 Ghent, Belgium

[1]Present address: Laboratory of Biochemistry and Brewing, Department of Applied Bioscience Engineering, Ghent University, Valentin Vaerwyckweg 1, 9000 Ghent

[2]Present address: Bioengineering Department, Faculty of Engineering, Ege University, 35100 Bornova-Izmir, Turkey

[*]Corresponding author:

Marjan De Mey

Phone: +32 9 264 60 28

Fax: +32 9 264 62 48

e-mail: Marjan.DeMey@UGent.be

Detailed, optimized protocol for the creation of a knock-out in *Citrobacter werkmanii* DSM17579

Linear double-stranded DNA The linear dsDNA amplicons were obtained by PCR using pKD3 (containing the chloramphenicol resistance cassette) and pKD4 (including the kanamycin resistance cassette) as templates and the high-fidelity PCR Master (Roche, Belgium) as master mix. Twenty nucleotides of the primers (Table S2, P1 and P2 primers) were complementary to the template and flanked with 100 nt complementary to the gene to be deleted. The PCR products were PCR-purified (QIAquick PCR purification kit, Qiagen, Netherlands), digested with *Dpn*I (New England Biolabs, Bioké, Belgium), repurified from an agarose gel (QIAquick Gel extraction kit, Qiagen, Netherlands), and suspended in elution buffer (10 mM Tris-Cl, pH 8.5).

Making of electrocompetent cells and electroporation of the cells Cells grown overnight in 5 mL LB with the appropriate antibiotic were transferred to 25 mL LB with the appropriate antibiotic to have an initial OD_{600nm} of 0.05. After incubating this culture to OD_{600nm} 0.6, 10 mL of the cells were rested on ice for 30 min to stop the metabolism. Thereafter, the cells were washed with 45 mL ice-cold Milli-Q (MQ)-water, a first time, and 1 mL ice-cold MQ-water, a second time, to remove the salts. After resuspending the cells in 50 µL ice-cold MQ-water, plasmid (100 ng) or linear dsDNA (400 ng) was added. The mixture was then transferred to an electroporation cuvette and electroporation was done using a Gene Pulser TM (BioRad, Belgium) (200 Ω, 25 µFD, and 250 V). Thereafter, 1 mL LB was added to the cells, after which they were incubated for 1 h when plasmid DNA was electroporated or 3 h when linear DNA was applied, and spread onto LB-agar containing the appropriate antibioticum. The plates were incubated at the appropriate temperature until colonies were formed (typically 16 h).

Preparation of the strain to insert linear dsDNA The cells were grown without antibiotics at 37 °C to an OD_{600nm} of 0.6. After making the cells electrocompetent, they were transformed with pKD46-Gm. Thereafter, they were incubated for 1 h on an orbital shaker at 200 rpm at 30 °C, and spread onto LB-

agar containing 50 µg/mL gentamicin, respectively. The gentamicin-resistant colonies were selected and tested for the presence of pKD46-Gm by PCR.

Transformation of the strain with linear dsDNA The cells with pKD46-Gm were grown with gentamicin (50 µg/mL) and 20 µM L-arabinose on an orbital shaker at 200 rpm at 30 °C to an OD_{600nm} of 0.6. After making the cells electrocompetent, they were transformed with linear dsDNA. Thereafter, the cells were incubated on an orbital shaker at 200 rpm at 37 °C for 3 h, and spread onto LB-agar containing 25 µg/mL chloramphenicol (when the linear DNA was amplified from pKD3) or 50 µg/mL kanamycin (when it was amplified from pKD4). The resistant colonies were verified by PCR with control primers on the homology regions (Table S2, control primers).

Deletion of the antibiotic marker The selected mutants (chloramphenicol- or kanamycin-resistant) were grown on an orbital shaker at 200 rpm at 37 °C to an OD_{600nm} of 0.6. Then, they were made electrocompetent and transformed with pCP20-Gm. The cells were again grown for 1 h on an orbital shaker at 200 rpm at 30 °C, and spread on LB-agar containing 50 µg/mL gentamicin. The resistant colonies were selected and tested by PCR using the control primers and by sequencing (LGC Genomics, Germany).
